# Rhizospheric and Endophytic Microbial Communities Associated with *Leptadenia pyrotechnica* in Arid Zones

**DOI:** 10.3390/microorganisms13091994

**Published:** 2025-08-27

**Authors:** Laila A. Damiati

**Affiliations:** Department of Biological Sciences, Collage of Science, University of Jeddah, Jeddah 21589, Saudi Arabia; ladamiati@uj.edu.sa

**Keywords:** metagenomics, microbial diversity, *Leptadenia pyrotechnica*, rhizosphere and endosphere, desert microbiome, 16S rRNA sequencing

## Abstract

Desert plants host specialized microbiomes that contribute to their survival under extreme conditions; yet, niche and specific microbial dynamics remain poorly understood. In this study, we used 16S rRNA amplicon sequencing to characterize the bacterial communities associated with *Leptadenia pyrotechnica*, which is a desert-adapted shrub. Five representative sample types were analyzed: rhizospheric soil from a non-arid adjacent location (control; S1); rhizospheric soil from the arid site (S4); and stem endosphere from the arid site (S5, S6, and S7). For each sample type, three biological replicates were collected from different healthy plants to ensure independence. Sequencing yielded high-quality datasets (89,000–134,000 reads/sample) with ASV retention ratios of 68–80%, confirming their sufficient depth for diversity profiling. Alpha diversity indices revealed a markedly greater richness in rhizospheric samples (e.g., S1 Shannon: 3.04; 530 ASVs) than in endosphere samples (Shannon < 1.0; ASVs ≤ 33), consistent with known gradients in desert plant microbiomes. Rarefaction curves confirmed the completeness of sampling. Beta diversity analyses, including PCoA and hierarchical clustering, showed clear segregation between rhizospheric and endophytic communities, indicating strong compartment-specific structuring. The rhizosphere was dominated by Actinobacteria (48%), Proteobacteria (32%), and Firmicutes (10%), whereas the stem endosphere was enriched in Proteobacteria (45%) and Actinobacteria (40%). Taxonomic profiling revealed that Bacillota and Actinomycetota dominated rhizospheric soils, including *Bacillus licheniformis*, while stem tissues were enriched in Cyanobacteriota and Alphaproteobacteria, suggesting host-driven filtering. Genera such as *Cupriavidus*, *Massilia*, and *Noviherbaspirillum* were exclusive to the rhizosphere, while *Paracholeplasma* appeared uniquely in stem sample S6. Archaea and rare phyla were nearly absent. The current findings indicate that *L. pyrotechnica* harbors distinct microbial assemblages in rhizospheric and endophytic niches, reflecting microhabitat-driven selection. These microbial communities may contribute to host resilience by harboring taxa with potential stress-tolerance traits, offering insights for microbiome-informed strategies in arid land restoration.

## 1. Introduction

*Leptadenia pyrotechnica* (Forssk.) Decne, commonly referred to as Khimp or Khip, is a xerophytic shrub belonging to the Apocynaceae family, and is native to arid regions spanning from the Thar Desert to the Sahel and Mediterranean zones of Africa and Asia [1]. Its extensive root system contributes significantly to sand dune stabilization, positioning it as a key pioneering species in desert afforestation programs [2]. Beyond its ecological role, *L. pyrotechnica* holds notable ethnobotanical importance, with various plant parts traditionally used to manage conditions such as infections, diabetes, fever, and wounds. These applications are supported by its documented antimicrobial, antioxidant, immunomodulatory, and anticancer activities [1].

In addition to its medicinal and ecological value, *L. pyrotechnica* harbors a diverse assemblage of microbial communities that may enhance its ability to withstand extreme desert conditions. Studies on isolating endophytic bacteria from their tissues have identified strains with plant growth-promoting traits, contributing to enhanced stress resilience and nutrient acquisition [3]. Seasonal metagenomic investigations have also revealed dynamic shifts in the composition of its rhizospheric microbiome, influenced by environmental factors such as soil moisture and temperature [4]. These findings are consistent with broader patterns observed in desert flora, where drought-adapted microbial taxa, particularly members of Actinobacteria and Proteobacteria, play crucial roles in promoting host plant survival under resource-limited conditions [5,6].

Advancements in cultivation-independent, high-throughput sequencing technologies have significantly improved our understanding of plant-associated microbiomes. These methods have enabled fine-scale characterization of microbial taxa and their functional attributes across distinct plant compartments, from the rhizosphere to internal tissues. Recent metagenomic studies in arid environments have demonstrated that rhizosphere communities are typically enriched with copiotrophic bacteria involved in carbon degradation and nitrogen cycling, while endophytic communities tend to exhibit narrower functional niches, reflecting specialized host–microbe interactions [7]. These microbiomes not only support plant health but also contribute to broader ecosystem functions, including nutrient turnover and stress tolerance.

Notably, several desert-associated microbes have demonstrated biosynthetic capabilities that may be employed for the development of bioactive materials and antimicrobial agents. These traits hold considerable promise for advancing tissue engineering approaches, especially in designing infection-resistant scaffolds and wound dressings. For instance, secondary metabolites or extracellular polymers produced by resilient endophytes could be incorporated into biocompatible matrices to enhance cell proliferation while reducing pathogen colonization, thereby supporting tissue regeneration under compromised conditions [8,9,10].

While desert plant microbiomes have been studied for several species, there is limited information on the microbiome of *L. pyrotechnica*, particularly regarding both its rhizospheric and endophytic compartments in arid environments. The absence of such integrated studies limits our understanding of how microhabitat-driven selection shapes microbial communities in this ecologically and economically important desert shrub.

The present study aimed to characterize the taxonomic composition of endophytic bacterial communities in the rhizosphere and shoots of plants associated with *L. pyrotechnica*, and to assess differences in alpha and beta diversity among these compartments. The current study hypothesizes that (i) rhizospheric and endophytic compartments of *L. pyrotechnica* harbor distinct microbial communities shaped by microhabitat conditions, and (ii) these communities include taxa potentially adapted to extreme aridity, contributing to plant survival. To address the current gap in comprehensive microbiome profiling of *L. pyrotechnica*, this study employed 16S rRNA amplicon sequencing to investigate microbial communities across five distinct sample types, including rhizosphere soils and stem tissues. The primary aim was to characterize the taxonomic composition and structural dynamics of these microbial assemblages from the phylum to genus level. Specifically, this study seeks to identify bacterial endophytes inhabiting internal plant tissues, delineate rhizospheric microbial communities, and assess variations in microbial structure across different microhabitats. By comparing rhizospheric and endophytic niches, this study aims to identify patterns of microhabitat-driven community structuring and highlight microbial taxa potentially adapted to extreme desert conditions. These findings will contribute to a broader understanding of plant–microbe interactions in arid ecosystems and establish a baseline for future functional studies. In particular, subsequent research may focus on isolating key extremotolerant microbes and characterizing their metabolites or enzymes, which could hold potential applications in agriculture, environmental biotechnology, and, in the long term, the development of stable bioactive compounds for regenerative medicine.

## 2. Materials and Methods

### 2.1. Sample Collection and Experimental Design

**Figure 1:**
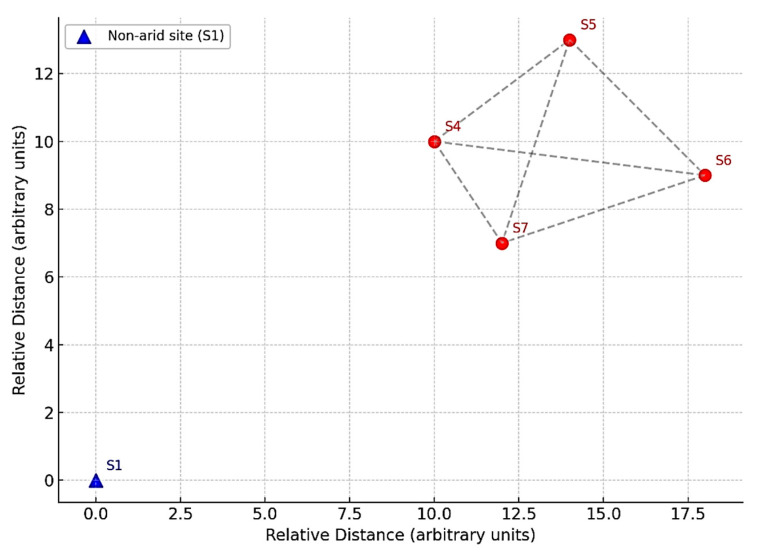
Sampling locations of Leptadenia pyrotechnica. Red markers (S4–S7) represent the arid desert site: S4 (rhizospheric soil) and S5–S7 (stem endosphere from three distinct shrubs). The blue marker (S1) represents the non-arid control site (rhizospheric soil). Coordinates correspond to the Al-Hejaz district of Jeddah, Saudi Arabia (21°47′20.9″ N, 39°15′58.2″ E). Distances are illustrated schematically to emphasize that individual shrubs were separated by at least 5 m to minimize spatial bias.

A total of five representative sample types were analyzed, each based on the average of three independent biological replicates. Healthy adult Leptadenia pyrotechnica shrubs (1.5–2 m in height) were sampled from two locations: (i) an arid desert site and (ii) a non-arid site serving as the control. In total, five shrubs were included in the study, and each was assigned a specific code: S1 (non-arid rhizospheric soil); S4 (arid rhizospheric soil); and S5, S6, and S7 (arid stem endosphere). For each shrub, three biological replicates were collected from the tissues or rhizospheric soil of the same individual plant, ensuring reproducibility and independence of measurements. Rhizospheric soil (S1 and S4) was obtained by gently uprooting the shrub and brushing off the soil adhering to the roots. Stem endosphere tissues were collected from three distinct shrubs at the arid site (S5, S6, and S7). Each replicate was processed separately after surface sterilization to ensure that only endophytic communities were analyzed. Non-consecutive sample codes reflected original field identifiers and were retained to preserve sequencing metadata integrity. Shrub selection followed strict criteria: (i) a healthy appearance without visible signs of disease or insect infestation; (ii) an adult growth stage (1.5–2 m height); (iii) naturally growing under undisturbed conditions; and (iv) a minimum spacing of 5 m between sampled shrubs to minimize spatial bias. All samples were collected under sterile conditions in the Al-Hejaz district of Jeddah, Saudi Arabia (21°47′20.9″ N, 39°15′58.2″ E), immediately transported on dry ice, and stored at −20 °C until DNA extraction. Figure 1 illustrates the two sampling sites and the approximate locations of the shrubs.

### 2.2. DNA Extraction and 16S rRNA Amplicon Sequencing

Genomic DNA was extracted from approximately 0.5 g of each sample using the DNAbler Cell and Tissue kit (Haven Scientific, Jeddah, Saudi Arabia), following the manufacturer’s instructions. DNA integrity was assessed via electrophoresis on 1% agarose gel, while purity and concentration were measured using a NanoDrop spectrophotometer (Thermo Fisher Scientific, Waltham, MA, USA). The V3–V4 hypervariable regions of the 16S rRNA gene were amplified using universal primers 341F (5′-CCTACGGGNGGCWGCAG-3′) and 806R (5′-GACTACHVGGGTATCTAATCC-3′), which target conserved bacterial regions and are widely used for taxonomic profiling. PCR amplification was performed according to standard protocols, and amplicon libraries were constructed using the Illumina 16S Metagenomic Sequencing Library Preparation workflow. Sequencing was carried out on the Illumina MiSeq platform using a 2 × 300 bp paired-end read configuration, generating high-quality reads for all samples. This approach enabled comprehensive coverage of the V3–V4 region for downstream diversity and taxonomic analyses.

### 2.3. Sequence Quality Control and ASV Processing

Raw sequence data were processed using a standardized quality control pipeline within the QIIME 2 environment (version 2023.2) (https://qiime2.org, accessed on 21 May 2025). Initially, sequencing adapters and primer sequences were removed using the cutadapt plugin (https://library.qiime2.org/plugins/q2-cutadapt, accessed on 21 May 2025). Subsequent preprocessing steps included length trimming and the removal of low-quality base calls to ensure uniformity across reads. Quality filtering was applied based on Phred scores, using either default or user-defined thresholds, to retain high-quality reads. Denoising of forward and reverse reads was conducted separately using the DADA2 algorithm (https://library.qiime2.org/plugins/q2-dada2, accessed on 21 May 2025), and this also corrected sequencing errors and resolved true biological sequences as amplicon sequence variants (ASVs). Paired-end reads were merged to reconstruct the full V3–V4 region of the 16S rRNA gene. Chimeric sequences were detected and removed using consensus-based chimera detection integrated within the DADA2 workflow. Following merging and chimera filtering, ASVs were further filtered based on sequence length and quality metrics to ensure that only reads with high confidence are included in downstream analyses. This rigorous sequence processing workflow enabled accurate delineation of microbial community composition with high taxonomic resolution.

### 2.4. Taxonomic Assignment and Microbial Profiling

Taxonomic classification of ASVs was performed using the QIIME 2 feature-classifier plugin (https://qiime2.org, accessed on 21 May 2025) with a Naive Bayes classifier trained on the SILVA 138 SSURef NR99 reference database (https://www.arb-silva.de, accessed on 21 May 2025), specific to the 16S rRNA V3–V4 region targeted during sequencing. ASVs were assigned across hierarchical taxonomic levels, including the phylum, class, order, family, genus, and species. The classification confidence threshold was set at 0.7 to ensure accurate taxonomic resolution. Following classification, relative abundance tables were generated and summarized at the phylum, genus, and species levels using the taxa package (https://cran.r-project.org/package=taxa, accessed on 21 May 2025) and phyloseq package (https://joey711.github.io/phyloseq, accessed on 21 May 2025) in R (https://www.r-project.org, accessed on 21 May 2025). These taxonomic profiles were used to compare the composition and structure of microbial communities across different compartments. Visualization of relative abundance distributions was carried out using ggplot2 (v3.4.2) (https://ggplot2.tidyverse.org, accessed on 21 May 2025), allowing for stacked bar charts, taxonomy plots, and differential distribution assessments. This workflow enabled high-resolution microbial profiling and comparative analyses of community composition across ecological niches.

### 2.5. Diversity Analyses

Alpha diversity analyses were conducted to assess microbial richness, evenness, and phylogenetic breadth within individual samples. The metrics calculated included the Shannon diversity index, which accounts for both the richness and evenness of taxa; the Gini–Simpson index, which reflects the probability that two randomly selected individuals belong to different species; Faith’s phylogenetic diversity (PD for the whole tree), which quantifies the total branch length of the phylogenetic tree connecting all taxa present in a sample; and the observed number of ASVs, representing species-level richness. These indices were computed using the diversity plugin in QIIME 2 (version 2023.2) (https://qiime2.org, accessed on 21 May 2025). To evaluate the sufficiency of sequencing depth and capture microbial richness across samples, rarefaction analyses were performed by generating curves for each diversity metric. Curves were plotted using QIIME 2 and visualized in R (https://www.r-project.org, accessed on 21 May 2025) with the ggplot2 package (v3.4.2) (https://ggplot2.tidyverse.org, accessed on 21 May 2025), ensuring that diversity estimates were not biased by unequal sequencing depth and that observed community differences reflected true biological variation rather than sampling artifacts.

### 2.6. Beta Diversity and Ordination Analysis

Distance Matrix: Pairwise dissimilarity values were computed using metrics such as Bray–Curtis dissimilarity or UniFrac distances to assess compositional differences among microbial communities. These calculations were performed using the QIIME 2 platform (https://qiime2.org, accessed on 21 May 2025), which supports both weighted and unweighted UniFrac methods based on phylogenetic information (https://library.qiime2.org, accessed on 21 May 2025). Principal Coordinates Analysis (PCoA): This multivariate ordination method was employed to visualize microbial community structure across samples. The analysis was conducted in QIIME 2, and the resulting plots were visualized in R (https://www.r-project.org, accessed on 21 May 2025) using the ggplot2 package (v3.4.2) (https://ggplot2.tidyverse.org, accessed on 21 May 2025). The first principal coordinate axis accounted for approximately 90% of the total variance, indicating strong compartmentalization. Hierarchical Clustering (Dendrogram): A phylogenetic dendrogram was constructed using QIIME 2 to depict relationships between microbial communities based on taxonomic or phylogenetic similarity. The resulting tree highlighted a clear separation between rhizospheric soil and stem-associated microbiomes.

### 2.7. Heatmap Visualization

Heatmaps were generated to visualize the relative abundance of microbial taxa across all five samples at the phylum, genus, and species levels. Abundance values were log_10_-transformed to enhance contrast and facilitate interpretation of taxonomic distribution patterns. Heatmaps were created using the heatmap package (version 1.0.12) (https://cran.r-project.org/package=pheatmap, accessed on 21 May 2025) and the ComplexHeatmap package (version 2.16.0) (https://jokergoo.github.io/ComplexHeatmap-reference/book/, accessed on 21 May 2025) in R (https://www.r-project.org, accessed on 21 May 2025). Taxonomic matrices used for heatmap construction were generated from the ASV table and aggregated at desired taxonomic levels (phylum, genus, species) using the phyloseq package (v1.44.0) (https://joey711.github.io/phyloseq, accessed on 21 May 2025). Color intensity in the heatmaps reflected log-transformed relative abundance values, and customized clustering, annotation, and color scaling were applied to optimize visualization. This approach enabled high-resolution, compartment-specific comparisons of microbial community composition across samples.

### 2.8. Statistical and Bioinformatic Tools

All bioinformatic analyses were conducted using a combination of QIIME 2 (version 2023.2) (https://qiime2.org) and R (version 4.3.1) (https://www.r-project.org) to ensure high-resolution processing, taxonomic assignment, and statistical interpretation of the 16S rRNA amplicon sequencing data. Initial sequence quality control, denoising, and chimera filtering were performed using the DADA2 plugin in QIIME 2 (https://library.qiime2.org/plugins/q2-dada2), which generated ASVs with single-nucleotide resolution. Taxonomic classification of representative ASVs was performed using a pre-trained Naive Bayes classifier against the SILVA 138 SSURef NR99 reference database (https://www.arb-silva.de), specific to the V3–V4 hypervariable region of the 16S rRNA gene. Alpha diversity indices, including Shannon’s index, the Gini–Simpson index, Faith’s phylogenetic diversity, and observed ASVs, were computed using the QIIME 2 diversity plugin (https://library.qiime2.org/plugins/q2-diversity). Beta diversity metrics based on Bray–Curtis, weighted UniFrac, and unweighted UniFrac distances were also calculated. Rarefaction analyses were performed to assess sequencing depth sufficiency. To further explore microbial community structure, Principal Coordinates Analysis (PCoA) and hierarchical clustering (UPGMA) were conducted in QIIME 2. Complementary data visualization and statistical analyses were carried out in R using the following packages: phyloseq (v1.44.0) (https://joey711.github.io/phyloseq) for microbiome data handling and visualization; vegan (v2.6-4) (https://cran.r-project.org/package=vegan) for ecological statistics; and ggplot2 (v3.4.2) (https://ggplot2.tidyverse.org) for graphical visualization. Heatmaps of log-transformed taxonomic abundances were generated using pheatmap (v1.0.12) (https://cran.r-project.org/package=pheatmap) and ComplexHeatmap (v2.16.0) (https://jokergoo.github.io/ComplexHeatmap-reference/book/). Phylogenetic tree construction and editing were supported using the ape package (v5.7-1) (https://cran.r-project.org/package=ape). Final data export and summary tabulations were performed using Microsoft Excel 365 and RStudio (version 2023.06.1+524) (https://posit.co/products/open-source/rstudio/). This integrative workflow ensured rigorous quality control, accurate taxonomic resolution, and robust visualization of microbiome patterns across different plant compartments. Data from all platforms were accessed during the period of 21–29 May 2025.

## 3. Results

### 3.1. Sequencing Quality Control and ASV Retention

16S rRNA gene amplicon sequencing of ten distinct sample types collected from *L. pyrotechnica* plants (including rhizosphere soil, stem tissues, and control soil) generated a comprehensive dataset of microbial diversity. For S1, the raw data consisted of 168,433 reads. After adapter and primer trimming, the read count was reduced to 166,565. Subsequent preprocessing and length trimming maintained this number at 166,565 reads (Table 1). Quality filtering further reduced this count to 143,423 reads, corresponding to a QC retention ratio of approximately 0.85. Denoising yielded 141,083 forward and 142,049 reverse reads. After merging, 133,917 paired reads were obtained and, from these, 113,999 reads were classified as non-chimeric. Finally, after ASV length filtering, 113,999 reads remained, achieving an ASV retention ratio of 0.68. For S4, the initial raw data had 162,585 reads, which decreased to 160,837 after adapter and primer trimming. This number remained stable after preprocessing and length trimming at 160,837. Quality filtering resulted in 136,755 reads (with a QC retention ratio of 0.84). Denoising produced 136,107 forward and 136,349 reverse reads, with merging generating 134,765 paired reads. Among these, 117,988 reads were non-chimeric, and following ASV filtering, 117,983 reads remained (with an ASV retention ratio of 0.73). S5 began with 120,770 raw reads, which were reduced to 119,255 after adapter and primer trimming and preprocessing. Quality filtering decreased reads to 94,873 (with a QC retention ratio of 0.79). Denoising resulted in 94,743 forward and 94,794 reverse reads, while merging yielded 94,689 pairs, with 89,304 reads identified as non-chimeric and remaining unchanged after ASV filtering (with an ASV retention ratio of 0.74). For S6, from the initial 142,409 raw reads, adapter trimming left 140,769 reads, and preprocessing maintained this count. Quality filtering produced 120,196 reads (with a QC retention ratio of 0.84). Post-denoising, 120,016 forward and 120,094 reverse reads were obtained, while merging yielded 119,935 pairs, and 113,318 reads were non-chimeric and unchanged by ASV filtering (with an ASV retention ratio of 0.80). S7 started with 172,119 raw reads, which were reduced to 170,168 by trimming and preprocessing. Quality filtering decreased the reads to 139,492 (with a QC retention ratio of 0.81). Denoising yielded 139,330 forward and 139,399 reverse reads, with merging resulting in 139,219 pairs. Of these, 133,133 were non-chimeric and remained after ASV filtering (with an ASV retention ratio of 0.77).

### 3.2. Taxonomic Profiling

**Figure 2:**
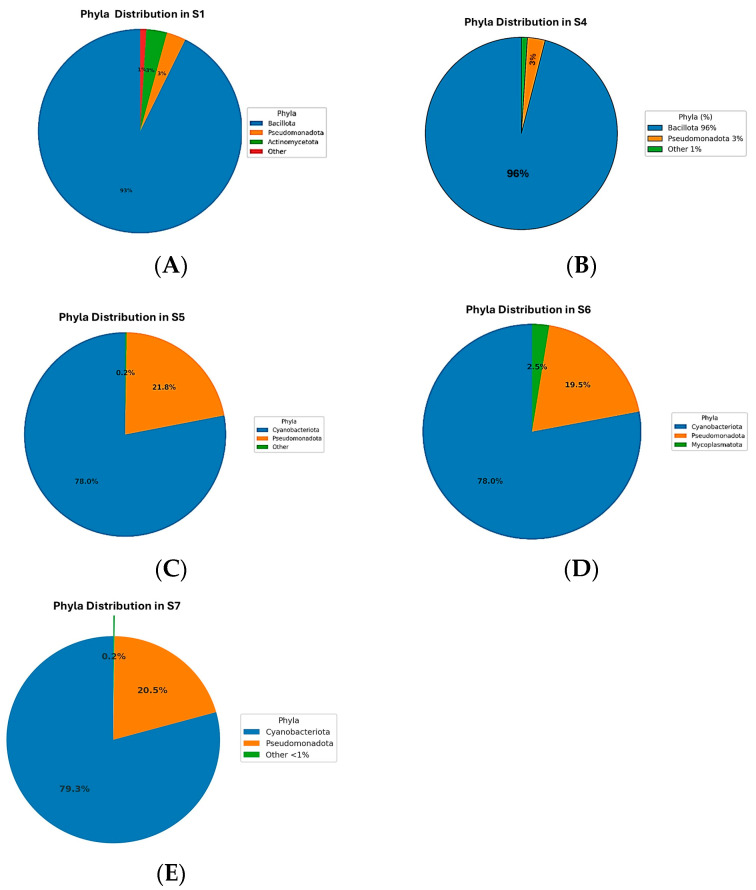
Phylum-level distribution of bacterial communities across five samples. Samples S1 (**A**) and S4 (**B**) were dominated by Bacillota (90.11% and 96.21%, respectively) with minor Actinomycetota. In contrast, Cyanobacteriota predominated in S5 (**C**), S6 (**D**), and S7 (**E**) 79.3%, accompanied by Pseudomonadota (up to 20. 5%), while Mycoplasmatota appeared uniquely in S6 (2.54%). Minor phyla such as Bacteroidota, Gemmatimonadota, Acidobacteriota, and Deinococcota were mainly detected in S1 and S4.

The microbiome analysis across five samples (S1, S4, S5, S6, S7) revealed distinct bacterial phyla distributions (Figure 2). The dominant phylum in S1 and S4 was Bacillota, constituting 90.11% and 96.21%, respectively (Figure 2A,B). In contrast, Cyanobacteriota was predominant in S5, S6, and S7, with relative abundances of approximately 78%. Pseudomonadota was consistently represented across all samples, ranging from 2.97% to 21.75%, and was particularly notable in S5, S6, and S7 (Figure 2C–E). Actinomycetota had notable representation only in S1 (3.17%) and S4 (0.57%). Mycoplasmatota uniquely appeared in S6 at 2.54%. Minor phyla such as Bacteroidota, Gemmatimonadota, Acidobacteriota, and Deinococcota had low abundances, predominantly in S1 and S4. The remaining phyla, including Myxococcota, Chloroflexota, Verrucomicrobiota, Deferribacterota, Planctomycetota, Thermomicrobiota, Bdellovibrionota, Rhodothermota, Armatimonadota, and Abditibacteriota, were detected at very low abundances or were nearly absent across samples. These findings highlight significant microbial community variations among the samples, indicating potential ecological differences influencing microbial distribution.

### 3.3. Genus-Level Microbial Composition

**Figure 3:**
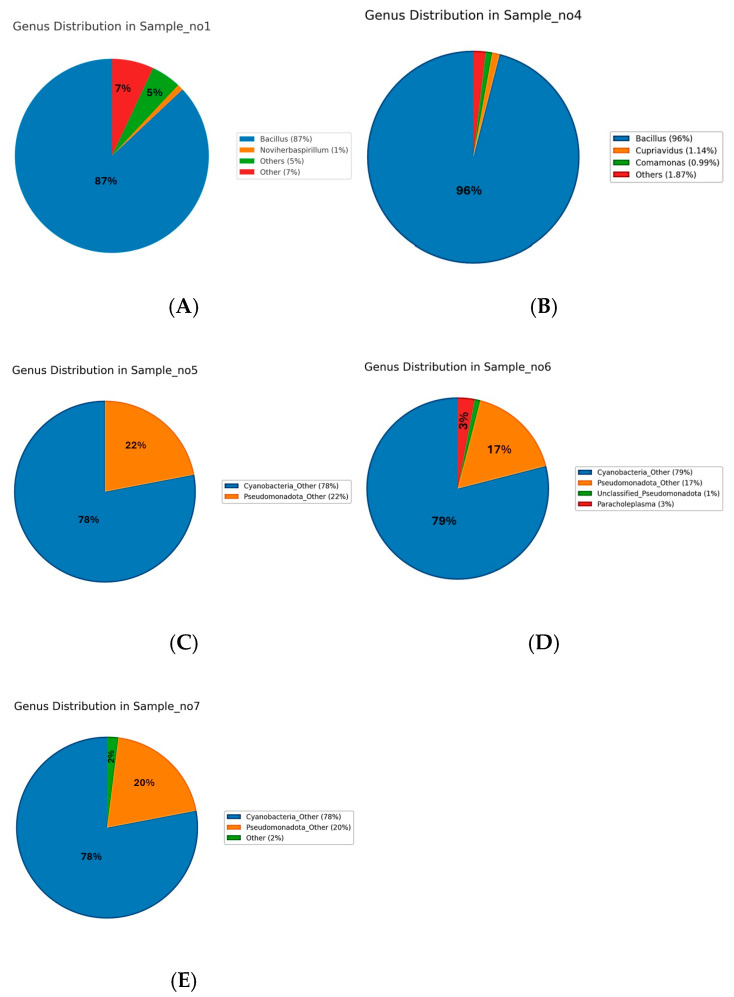
Genus-level relative abundance of microbial communities in samples S1–S7. In the rhizospheric samples, S1 (**A**) and S4 (**B**) were strongly dominated by *Bacillus* (87% and 96.00%, respectively), with minor contributions from *Cupriavidus*, *Noviherbaspirillum*, and other genera, each generally contributing less than 1%. In contrast, the endophytic samples S5 (**C**)**,** S6 (**D**), and S7 (**E**) were also dominated by *Cyanobacteria* (78–79%), *Pseudomonadota* (~20%), with the remining of the community consisting of taxa at ~2%.

At the genus level, distinct microbial community profiles were observed across the five samples (Figure 2A–E). In the rhizospheric samples, S1 (Figure 3A) was dominated by *Bacillus*, which accounted for approximately 87% of the total sequences, followed by minor proportions of *Noviherbaspirillum* (1%), *Massilia* (0.9%), *Cupriavidus* (0.75%), *Kocuria* (0.77%), and *Microvirga* (0.71%), with the remaining genera each contributing less than 1% and grouped as “Other 5 or 7%)”. Similarly, S4 (Figure 3B) exhibited a pronounced predominance of *Bacillus* (96%), accompanied by *Cupriavidus* (1.14%), *Comamonas* (0.99%), with all other genera present below 2%. In contrast, the endophytic compartments displayed a markedly different taxonomic structure. S5 (Figure 3C) was dominated by unclassified *Cyanobacteria* (78%) and unclassified *Pseudomonadota* (22%), with no other genera exceeding the 1% threshold. S6 (Figure 3D) followed a similar pattern, with unclassified *Cyanobacteria* (79%) and *Pseudomonadota* (17%) prevailing, alongside the unique detection of *Paracholeplasma* (3%) and all other genera at <1%. Likewise, S7 (Figure 3E) was composed primarily of unclassified *Cyanobacteria* (78%) and *Pseudomonadota* (20%), with the remainder of the community consisting of taxa at 2%. Collectively, these results reveal a clear compartment-specific trend, with *Bacillus* dominating in rhizospheric communities and unclassified *Cyanobacteria* together with *Pseudomonadota* prevailing in endophytic niches.

### 3.4. Species-Level Microbial Composition

**Figure 4:**
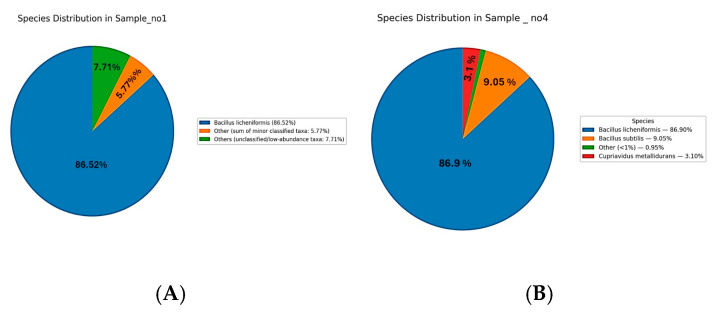

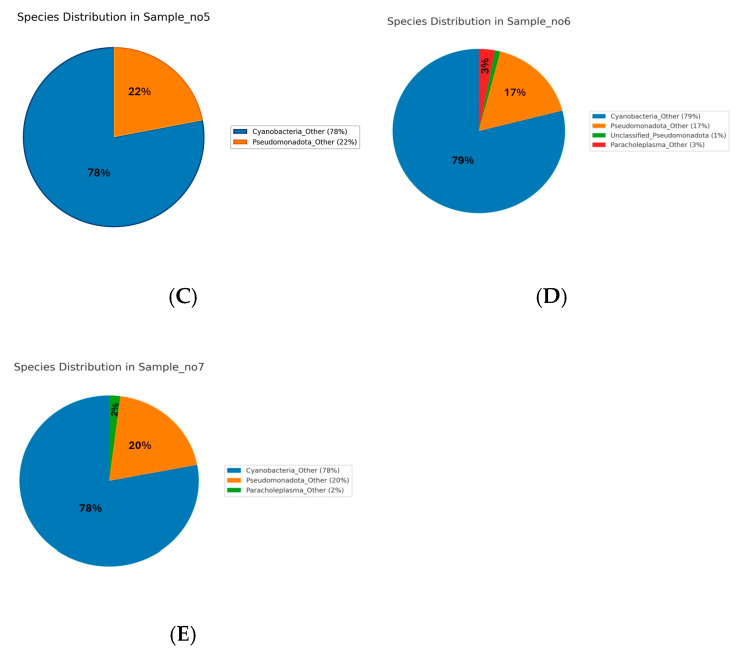
Species-level microbial composition of samples S1–S7. Rhizospheric samples S1 (**A**) and S4 (**B**) were dominated by *Bacillus licheniformis* (86.5% and 86.9%, respectively), with minor contributions from *Cupriavidus metallidurans*, *Bacillus subtillus*, and other low-abundance taxa (<1%). Endophytic samples S5–S7 (**C**–**E**) were instead dominated by unclassified *Cyanobacteria* (78–79%) and *Pseudomonadota* (17–22%), with additional minor taxa such as *Paracholeplasma* (2–3%) and unclassified *Pseudomonadota* (1%).

The species-level analysis of microbial communities across Samples S1 to S7 revealed distinct patterns of dominance (Figure 4). In Sample S1, *Bacillus licheniformis* was overwhelmingly dominant, representing 86.5% of the community. Minor species included *Cupriavidus metallidurans* (0.75%), *Metabacillus niabensis* (0.54%), *Massilia horti* (0.46%), *Noviherbaspirillum aridicola* (0.36%), *Priestia koreensis* (0.35%), *Arthrobacter flavus* (0.22%), and others each contributing less than 1%, while the remaining taxa were grouped as “Others” (7.7%) (Figure 4A). In S4, *Bacillus licheniformis* also dominated at 86.9%, followed by *Bacillus subtilis* (9.05%) and *Cupriavidus metallidurans* (3.1%), with minor contributions from other species (Figure 4B). In S5, the community was dominated by unclassified *Cyanobacteria* species (78%) and *Pseudomonadota* species (22%) (Figure 4C). S6 followed a similar pattern, with Cyanobacteria (79%) and Pseudomonadota (17%) as the main taxa, along with a unique contribution from unclassified Paracholeplasma species (3%), and minor “unclassified” (1%) (Figure 4D). In S7, *Cyanobacteria* (78%) and *Pseudomonadota* (22%) dominated the microbial profile (Figure 4E). Across samples S5 to S7, the identified species-level diversity was low, with most taxa grouped as unclassified or grouped as “Other”.

### 3.5. Heat Map at Phylum Level

**Figure 5:**
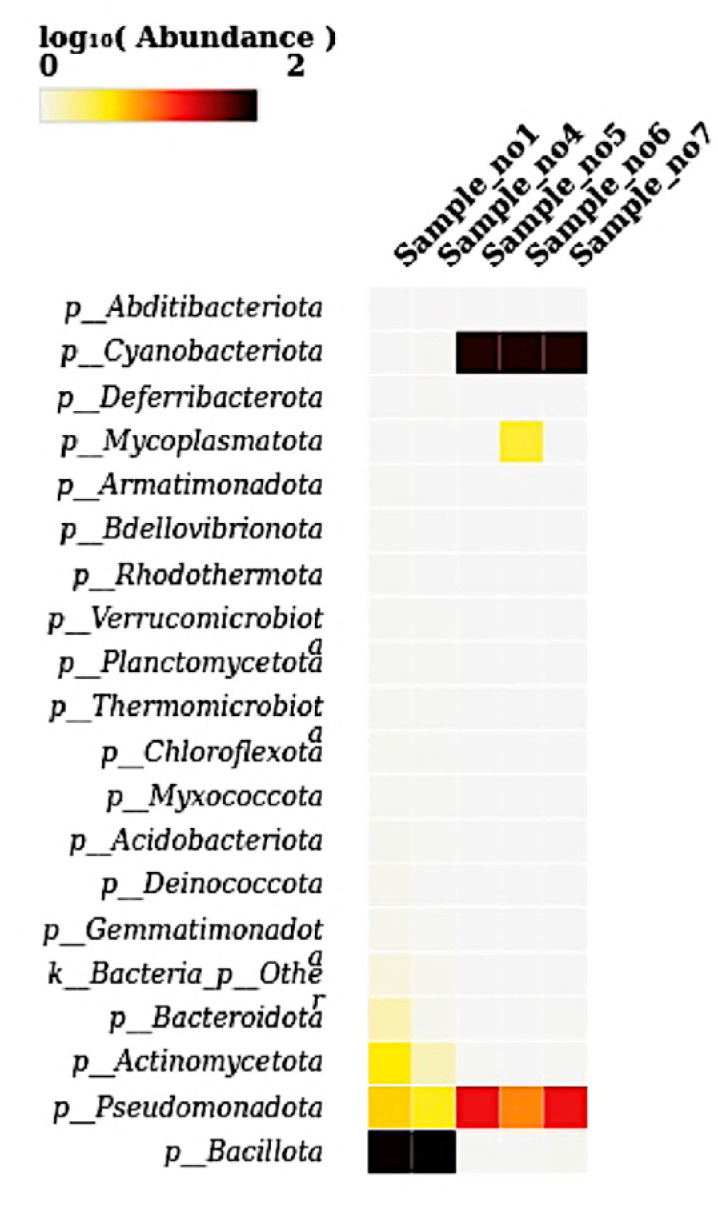
A heatmap showing the relative abundance (log_10_-transformed) of dominant bacterial phyla across the five samples (S1 to S7). The heatmap illustrates phylum-level taxonomic distribution, with intensity representing the log-transformed abundance values. Notably, Bacillota and Pseudomonadota exhibited high abundances in S1 and S4, while Cyanobacteriota dominated S5, S6, and S7. Additionally, Mycoplasmatota was detected at elevated levels specifically in S6. Several low-abundance phyla, such as *Planctomycetota*, *Acidobacteriota*, and *Bdellovibrionota,* were sparsely distributed across all samples.

The heatmap visualizes the microbial abundance (log10-transformed) across the five analyzed samples, highlighting specific patterns. Bacillota showed very high abundance in S1 and S4, indicated by intense dark coloration. Cyanobacteriota displayed similarly high abundances in S5, S6, and S7. Pseudomonadota demonstrated moderate abundance across all samples, which is especially notable in S5, 6, and 7 (Figure 5). Mycoplasmatota was distinctly abundant only in S6. Actinomycetota, Bacteroidota, and the “Other” bacterial phylum exhibited lower but detectable levels in S1 and S4. The remaining phyla were present at very low or undetectable levels, depicted by minimal or no coloration in the heatmap.

### 3.6. Heatmap at Genus Level

**Figure 6:**
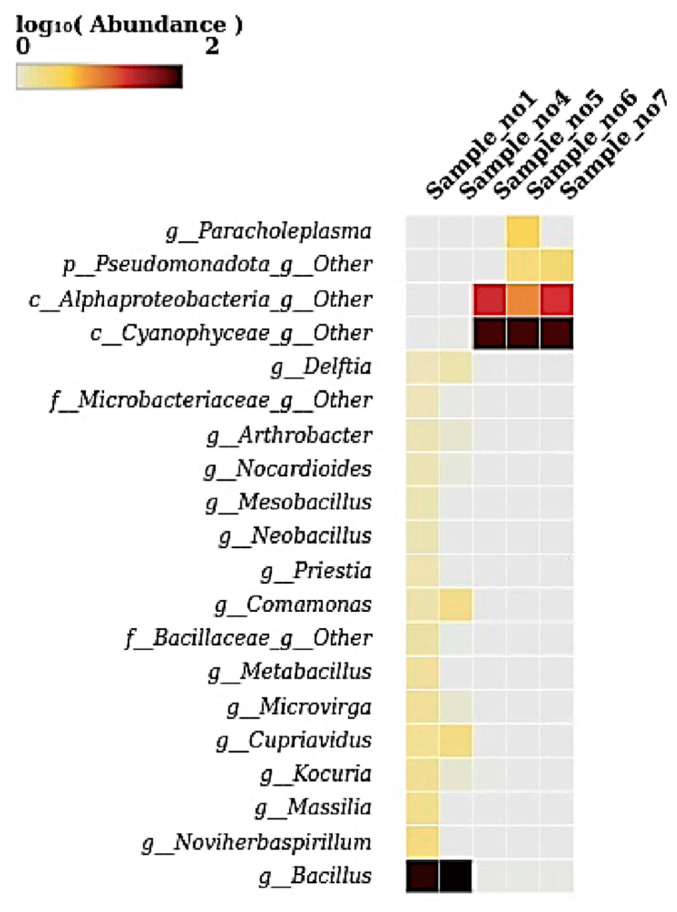
A heatmap showing the relative abundance (log_10_-transformed) of bacterial genera across the five samples (S1 to S7). This heatmap visualizes the distribution of key bacterial genera across samples, with color intensity corresponding to log-transformed abundance. *Bacillus* was highly abundant in S1 and S4, while unclassified members of *Cyanophyceae* and *Pseudomonadota* were dominant in S5, S6, and S7. The genus *Paracholeplasma* was notably enriched in S6, while genera such as *Cupriavidus*, *Kocuria*, *Noviherbaspirillum*, and *Microvirga* appeared at low abundances across the datasets. These patterns reflect distinct shifts in microbial communities among the samples.

The heatmap illustrates the log10-transformed abundance of microbial taxa at the genus level across five samples. The genus Bacillus displayed high abundance in S1 and S4, which is evident from the intense dark color. The genus from the class Cyanophyceae was abundant in S5, S6, and S7. Genera from Alphaproteobacteria and Pseudomonadota showed moderate abundance in these samples (S5, S6, and S7) (Figure 6). The genus Paracholeplasma was notably abundant only in S6. Other genera, such as Delftia, Comamonas, Microvirga, Cupriavidus, Massilia, and Noviherbaspirillum, exhibited lower yet detectable abundances primarily in S1 and S4. The remaining genera were present at very low or negligible levels, as indicated by faint or no coloration.

### 3.7. Heatmap at Species Level

**Figure 7:**
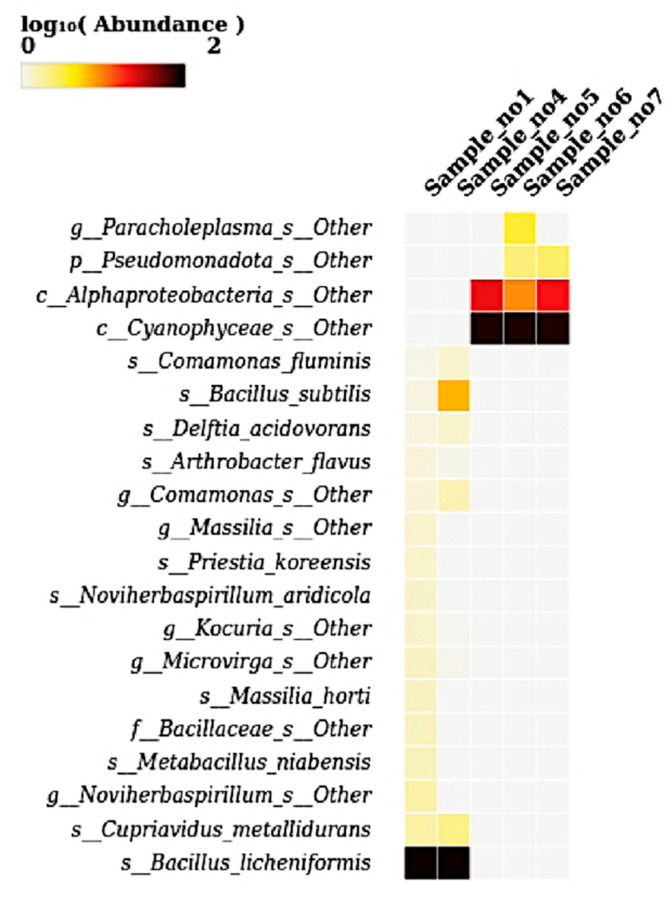
A heatmap showing the relative abundance (log_10_-transformed) of bacterial species across five samples (S1 to S7). The heatmap presents species-level taxonomic profiles based on log-transformed abundance values. *Bacillus licheniformis* exhibited dominant abundance in S1 and S4, while unclassified species of *Cyanophyceae* and *Alphaproteobacteria* were prevalent in S5, S6, and S7. *Paracholeplasma* species appeared uniquely in S6. Other low-abundance species such as *Cupriavidus metallidurans*, *Massilia horti*, *Priestia koreensis*, *Delftia acidovorans*, and *Noviherbaspirillum aridicola* were detected in S1 and S4. These variations reflect clear shifts in microbial community composition at the species level across the analyzed samples.

The heatmap illustrates the log_10_-transformed abundance of microbial species across five samples, revealing distinct compositional patterns. *Bacillus licheniformis* was highly abundant in S1 and S4, as indicated by the dark coloration, suggesting its dominance in these environments (Figure 7). Similarly, *Bacillus subtilis* and other Bacillus-related taxa such as *Delftia acidovorans*, *Comamonas fluminis*, and *Arthrobacter flavus* were moderately abundant in the same two samples. In contrast, S5, S6, and S7 were dominated by *Cyanophyceae_s__Other*, showing intense coloration, along with the moderate presence of *Alphaproteobacteria_s__Other* and *Pseudomonadota_s__Other*. Notably, *Paracholeplasma_s__Other* showed marked abundance specifically in S6. Other taxa, including *Priestia koreensis*, *Novihersbaspirillum aridicola*, *Microvirga*, *Kocuria*, *Cupriavidus metallidurans*, and *Metabacillus niabensis,* appeared at a lower abundance primarily in S1 and S4. Overall, the heatmap highlights a clear compositional divergence: Bacillus-dominated microbiota in the first two samples versus Cyanophyceae- and Proteobacteria-dominated communities in the latter three samples.

### 3.8. Taxonomy Abundance Ratio

Phylum-level microbial community analysis across the five samples (S1, S4, S5, S6, and S7) revealed notable shifts in bacterial dominance. In S1 and S4, Bacillota was the overwhelmingly dominant phylum, comprising 90.11% and 96.21% of the microbial community, respectively. These samples also showed a moderate presence of Pseudomonadota (5.69% and 2.97%) and minor contributions from Actinomycetota (3.17% and 0.57%). In contrast, S5, 6, and 7 were dominated by Cyanobacteriota, representing 78.26%, 78.67%, and 78.18% abundance, respectively. In the same samples, Pseudomonadota also exhibited substantial abundance at 21.71%, 18.78%, and 21.75%. A unique finding was the detection of Mycoplasmatota at 2.54% exclusively in S6. Other phyla, including Bacteroidota, Gemmatimonadota, Chloroflexota, and Deinococcota, appeared at low percentages (≤0.6%) mainly in S1 and S4, with negligible or absent presence in other samples. Archaea, such as Methanobacteriota, Nitrososphaerota, and Thermoplasmatota, were not detected in any sample. Overall, the data reflect a clear shift from Bacillota-dominated microbiota in S1 and S4 to Cyanobacteriota- and Pseudomonadota-dominated profiles in S5 through S7.

### 3.9. Alpha Diversity

**Figure 8:**
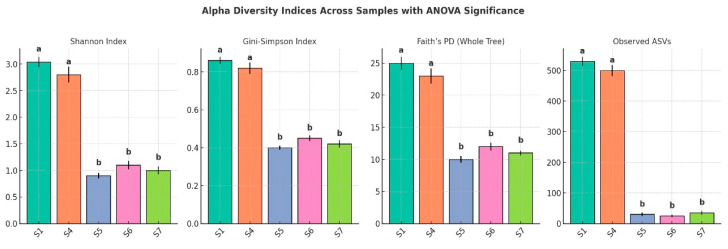
Alpha diversity indices across five sample types (S1–S7), including the Shannon index, Gini–Simpson index, Faith’s phylogenetic diversity (PD for the whole tree), and the number of observed amplicon sequence variants (ASVs). Error bars represent standard deviations (*n* = 3 biological replicates per sample type). The different letters above the bars indicate statistically significant differences among samples based on one-way ANOVA followed by Tukey’s HSD test (*p* < 0.05). Samples sharing the same letter were not significantly different.

The alpha diversity analysis across the five samples revealed a substantial variation in microbial community richness and evenness (Figure 8). S1 exhibited the highest diversity, with a Shannon index of 3.04, a Gini–Simpson index of 0.70, and a phylogenetic diversity (PD for the whole tree) value of 45.48. This sample also had the highest number of observed amplicon sequence variants (ASVs), totaling 530, indicating a significantly rich and phylogenetically diverse community. Sample_no4 followed with moderate diversity, showing a Shannon index of 1.66, a Gini–Simpson index of 0.54, a PD value of 29.25, and 190 ASVs. In contrast, S5, S6, and S7 demonstrated markedly lower diversity. S5, in particular, had the lowest Shannon index (0.78), the fewest ASVs (7), and the lowest PD value (2.80), suggesting a significantly limited microbial community. S6 and S7 showed slightly higher diversity than S5 but remained substantially less diverse than S1 and S4. ANOVA confirmed that these differences were statistically significant (*p* < 0.05), with post hoc tests grouping samples according to their shared diversity levels. Overall, diversity and richness declined sharply from the control rhizospheric soil (S1) to the endophytic compartments, underscoring strong niche-specific structuring of the *L. pyrotechnica* microbiome.

### 3.10. Rarefaction

The rarefaction analysis conducted using four alpha diversity indices—ASVs, Faith’s PD for the whole tree, Shannon’s Index, and the Gini–Simpson index—demonstrated substantial differences in microbial diversity across the five analyzed samples.

For observed ASVs, which reflect species-level richness, S1 exhibited the greatest richness, reaching a plateau at approximately 530 ASVs, followed by S4 with around 190 ASVs. In contrast, S5, S6, and S7 displayed low microbial richness, plateauing at 7, 13, and 33 ASVs, respectively. These patterns suggest that the microbial communities in S1 and S4 are significantly richer than those in the remaining samples.

Faith’s PD for the whole tree rarefaction curves, which incorporate evolutionary relationships among taxa, further confirmed this trend. S1 displayed the highest phylogenetic diversity (~45), indicating a community composed of taxa from a wide range of evolutionary lineages. Sample_no4 followed with a PD value of ~29. S7, S6, and S5 exhibited much lower values (~9, ~6, and ~3, respectively), signifying narrower phylogenetic breadth in their microbial communities.

The Shannon diversity index, which accounts for both richness and evenness, reinforced the observed trends. S1 had the highest Shannon value (~3.0), indicating not only high levels of richness but also a balanced distribution of taxa. S4 showed moderate diversity (~1.7), while S5, S6, and S7 had much lower values (~0.75–0.95), reflecting communities dominated by few taxa with low evenness.

The Gini–Simpson index, another measure of diversity and evenness, yielded comparable results. S1 ranked highest again (~0.70), followed by S4 (~0.54), with the remaining three samples clustering at the lower end of the scale (~0.34–0.35), further highlighting their limited diversity and dominance by fewer taxa.

Collectively, the rarefaction results demonstrate that S1 and S4 possess distinctly richer and more diverse microbial communities, both taxonomically and phylogenetically, compared to S5, S6, and S7. The clear plateauing of all curves confirms that sequencing depth was sufficient to capture most of the diversity within each sample.

**Figure 9:**
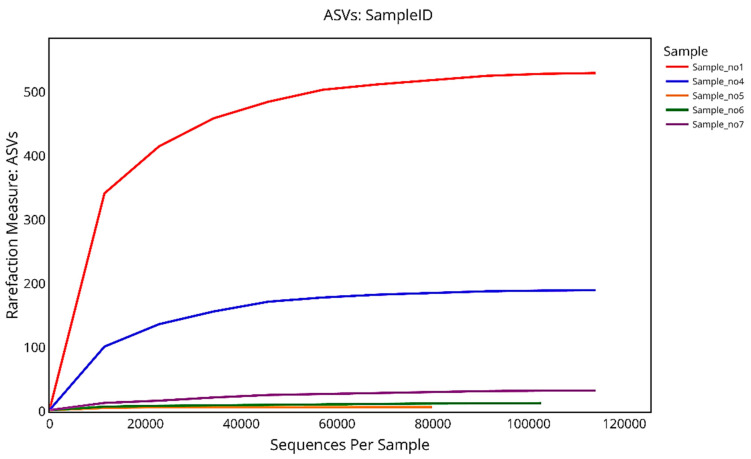
Rarefaction curves showing the number of observed ASVs (amplicon sequence variants) as a function of the sequencing depth for five samples. The curves illustrate microbial richness across samples, with S1 and S4 displaying the highest ASV counts and reaching clear plateaus, indicating sufficient sequencing depth. By contrast, S5, S6, and S7 exhibit low richness with early curve flattening, reflecting limited microbial diversity.

The rarefaction curve based on ASVs (amplicon sequence variants) illustrates the richness of microbial communities in relation to sequencing depth across five samples. S1 exhibited the highest species richness, with its curve sharply rising and eventually plateauing at around 530 ASVs, indicating sufficient sequencing depth to capture most of the diversity. S4 followed with a moderate increase, reaching approximately 190 ASVs. In contrast, S5, S6, and S7 showed very limited microbial richness, with their curves flattening out prematurely, reaching around 7, 13, and 33 ASVs, respectively. The plateauing of all curves confirms adequate sequencing coverage, while the differences in curve height reflect substantial variation in microbial richness among the samples (Figure 9).

### 3.11. β-Diversity

#### 3.11.1. Distance Matrix

The pairwise β-diversity analysis based on Bray–Curtis dissimilarity values revealed pronounced differences in microbial community composition across the five samples. Values ranged from 0 (identical communities) to 1 (completely dissimilar), with lower values reflecting greater similarity. S1 and S4 showed a moderate level of dissimilarity (0.455), indicating some overlap in their community composition but also notable differences. In contrast, S1 exhibited extremely high dissimilarity with S5 (0.9998), S6 (0.9999), and S7 (0.9996), suggesting near-complete differences in their microbial profiles. Similarly, S4 was highly dissimilar for S5 (0.9999), S6 (0.9999), and S7 (0.9998), reinforcing the idea that S1 and S4 harbor distinct microbial communities compared to the remaining samples. On the other hand, S5, S6, and S7 shared greater similarity among themselves, with dissimilarity values ranging from 0.10 to 0.20, indicating a more uniform and less diverse microbial structure. These trends are summarized in Table 2, which highlights the clear separation between the more diverse rhizospheric communities (S1, S4) and the more similar stem-associated communities (S5–S7).

#### 3.11.2. Phylogeny Tree (Dendrogram)

**Figure 10:**
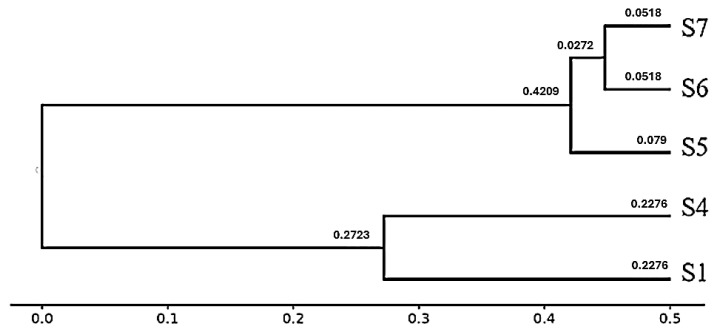
Hierarchical clustering dendrogram of microbial communities based on β-diversity distances. Two major clusters were identified: the first comprising S1 and S4 (rhizospheric samples), and the second comprising S5, S6, and S7 (endophytic samples). The numerical values shown on the branches represent pairwise dissimilarity scores, where smaller values indicate greater similarity and larger values indicate greater dissimilarity. Branch lengths correspond to β-diversity distances, clearly separating rhizospheric from endophytic microbial communities.

The hierarchical clustering dendrogram, constructed from β-diversity distance metrics, reveals two distinct clusters among the five samples. Samples S1 and S4 are grouped together, reflecting high similarity in their microbial community structures (pairwise distance = 0.2276). The second cluster comprises S5, S6, and S7, with S6 and S7 showing the closest relationship (distance = 0.0518), followed by their association with S5 (distances = 0.079 and 0.0272, respectively) (Figure 10). These patterns are consistent with the pairwise distance matrix and PCoA results, indicating that S1 and S4 harbor distinct and more diverse microbial communities compared to the similar and less diverse assemblages in S5–S7.

#### 3.11.3. The Principal Coordinates Analysis Plots

The Principal Coordinates Analysis (PCoA) plots reveal consistent and biologically meaningful patterns in microbial beta diversity across the five samples using different distance metrics. In all three plots, S1 and S4 are distinctly separated from the remaining samples, reflecting major compositional and structural differences in their microbial communities. Conversely, S5, S6, and S7 are consistently clustered close together, suggesting highly similar microbial profiles, which are likely characterized by lower diversity and shared dominant taxa.

**Figure 11:**
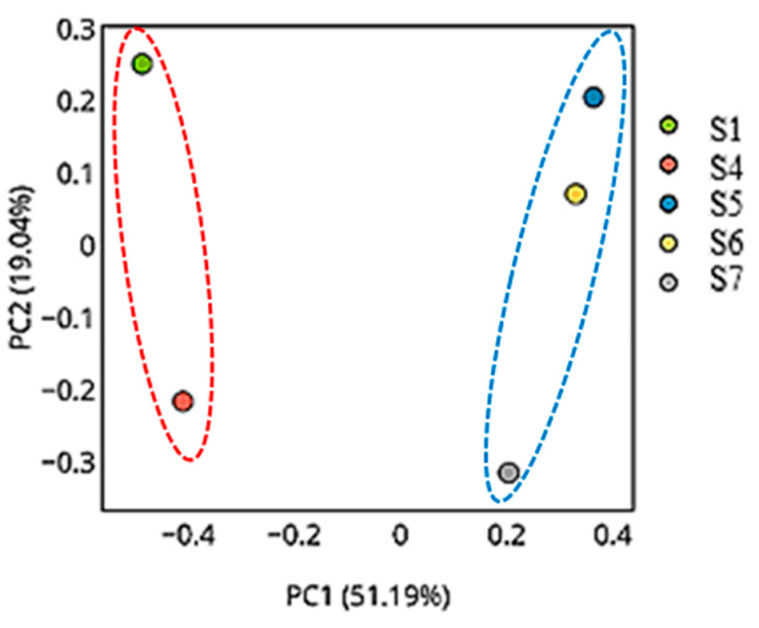
Principal Coordinates Analysis (PCoA) plot based on beta diversity distances (weighted UniFrac or Bray–Curtis dissimilarity), showing clear separation between high-diversity samples (S1 and S4) and low-diversity samples (S5, S6, and S7). PC1 explains 90.04% of the total variation, indicating that differences in taxonomic abundance and community structure are the primary source of diversity among samples. The red dotted circle highlights the rhizospheric samples (S1 and S4), while the blue dotted circle groups the endophytic samples (S5, S6, and S7), indicating distinct clustering of microbial communities by sample type.

In the PCoA plot (Figure 11), which explains the largest proportion of variation (PC1: 90.04%, PC2: 8.27%), a clear separation is evident along PC1 between the high-diversity group (S1 and S4) and the low-diversity group (S5 to S7). The dominance of this axis indicates that the main beta diversity differences are driven by differences in taxonomic abundance or community structure.

Overall, these PCoA plots demonstrate that microbial communities in S1 and S4 are compositionally distinct and more diverse, while S5, S6, and S7 form a homogeneous cluster with low diversity and minimal taxonomic differentiation. These patterns are consistent with alpha diversity indices, rarefaction results, and the pairwise distance matrix.

## 4. Discussion

The quality control (QC) outcomes of the 16S rRNA amplicon sequencing data provide strong evidence for dataset robustness and ecological validity. Retention rates after filtering ranged between ~81% and ~85% across all samples, while amplicon sequence variant (ASV) retention varied between ~68% and ~77%. The observed variability mirrors patterns documented in benchmarking studies, where the complexity of communities strongly influences ASV retention following denoising with DADA2 [11]. For instance, samples S1 and S4, both representing rhizospheric communities with significant species richness, showed slightly reduced ASV retention compared to endosphere samples with lower diversity (e.g., S7). This trend has been attributed to the algorithmic tendency to split reads more extensively in complex communities while achieving higher read assignments in less complex microbiomes [11]. Comparable retention rates have been reported in desert rhizosphere microbiome studies. For instance, a study on arid-zone legumes recorded a post-filtering retention of 75–88% of input reads and ASV yields consistent with the current findings [7]. Taken together, the QC metrics are consistent with established thresholds in environmental microbiome research, affirming the reliability of the dataset and suggesting that sample diversity influenced both read retention and ASV resolution in expected ways.

This study reveals the clear, niche, and specific compartmentalization of microbial communities associated with *L. pyrotechnica* from the Al-Hejaz district of Jeddah, with distinct taxonomic and structural differences between rhizospheric and stem microbiomes. Rhizosphere samples were dominated by Bacillota (>90%, mainly Bacilli), with lower proportions of Pseudomonadota and Actinomycetota, while stems were enriched in Cyanobacteriota (~78%) and Pseudomonadota, with Mycoplasmatota detected in one sample. Minor phyla such as Bacteroidota, Gemmatimonadota, Acidobacteriota, and Deinococcota occurred only in the rhizosphere. These patterns likely reflect abiotic and host-driven factors, such as the rhizosphere’s heterogeneous soil, nutrient-rich root exudates, and moisture retention, which support high diversity, favoring spore-forming and stress-tolerant Bacillota; in contrast, the nutrient-poor, immune-filtered stem endosphere contains specialized taxa such as Cyanobacteriota, capable of nitrogen fixation, osmoprotection, and photoprotection. These results align with desert plant microbiome studies that show richer and more functionally diverse belowground communities compared to internal tissues. In this study, rhizospheric samples showed higher richness and phylogenetic diversity (Shannon ~3.0; ASVs > 500) than stems (Shannon < 1.0; ASVs ≤ 33), paralleling findings in Senna italica and Alhagi sparsifolia, where rhizosphere and endosphere communities diverge sharply [12,13].

Rhizosphere microbiomes in arid ecosystems are often characterized by phyla associated with organic matter degradation, abiotic stress resistance, and plant growth promotion. The current results reflect this pattern, with Actinomycetota, Bacillota (notably *Bacillus licheniformis*), and Bacteroidota predominating in rhizospheric soil. These phyla have been previously reported in desert environments such as the Namib Desert, where they contribute to nutrient cycling and drought resilience [14]. *B. licheniformis* in particular has been identified as a potent plant growth-promoting bacterium in arid soils, producing extracellular enzymes and secondary metabolites beneficial to plant health [3]. Comparative studies on desert halophytes in Pakistan also support the current findings, where rhizosphere communities were dominated by Proteobacteria and Actinobacteria, while Cyanobacteria formed a major component of the stem endosphere, at times comprising up to 75% of the microbial community [15].

The dominance of Cyanobacteriota in the internal stem tissues of *L. pyrotechnica* suggests that these taxa may play ecologically relevant roles, as reported in other plant systems. Cyanobacteria have been documented in the literature to engage in nitrogen fixation and light-driven metabolic processes, which are traits that could be advantageous in the exposed, resource-limited stem tissues of desert plants [16]. The selective detection of Mycoplasmatota in only one stem sample is noteworthy, as members of this phylum are infrequently reported in healthy plant microbiomes; their occurrence here may reflect localized host selection or transient environmental colonization [17]. Additionally, the presence of rare taxa such as *Paracholeplasma*, found exclusively in one stem sample, is intriguing. While this study did not assess functional genes, previous research has indicated that such rare endophytes can harbor unique genetic traits, including potential bioactive compound biosynthesis or stress mitigation mechanisms. These possibilities warrant further investigation through targeted functional analyses.

The consistent detection of Pseudomonadota across all compartments is consistent with their widely reported ubiquity and ecological adaptability in plant microbiomes. Alphaproteobacteria, a key class within this phylum, have been described in previous studies as possessing plant-beneficial traits such as phytohormone synthesis and the production of osmoprotectants, which can enhance host stress tolerance [18]. Their occurrence in internal plant compartments, as observed here, may therefore have ecological relevance in arid environments, where osmotic balance and hormone regulation are important for plant survival. Reports from the literature indicate that some desert endophytes, including *Pseudomonas* spp., can improve abiotic stress tolerance in crops when introduced as inoculants; for instance, a *Pseudomonas* endophyte isolated from *A. sparsifolia* was shown to enhance drought tolerance in wheat under field conditions [13].

The pronounced beta-diversity separation observed here reflects more than just taxonomic turnover; it is consistent with ecological filtering processes along the soil–shoot continuum. In the rhizosphere (S1 and S4), microbes inhabit a nutrient-rich, microbially competitive environment at the soil–root interface, which selects metabolically versatile taxa capable of rapid colonization and competitive exclusion. In contrast, the shoot endosphere compartments examined in this study (S5, S6, and S7) represent highly selective niches within aboveground plant tissues, where microbial communities are shaped by host-mediated filtering, physiological constraints, and specialized adaptations for survival and persistence inside the plant. Within the shoots, structural barriers, antimicrobial compounds, and immune recognition systems impose a narrow entry gate, favoring a subset of taxa with traits for immune evasion, endophytic persistence, and metabolic compatibility with host physiology. This stepwise filtering process—common in desert and xerophytic plants—generates distinct, niche-adapted communities, as documented in *Alhagi sparsifolia* and *Tamarix ramosissima* [13].

Beta diversity analyses confirmed clear separation between rhizosphere and stem microbiomes. PCoA and hierarchical clustering consistently grouped rhizosphere samples apart from stem-associated ones, reflecting strong ecological differentiation, consistent with reports in desert legumes and xerophytes where environmental filters, compartment specificity, and microclimatic conditions shape microbial communities [4,13]. The enrichment of soil-specific phyla such as Acidobacteriota and Gemmatimonadota—which are slow-growing, oligotrophic taxa absent from internal tissues—highlights the rhizosphere’s ecological complexity and role in nutrient cycling [19].

Seasonal dynamics, driven by moisture, temperature, and phenology, may further influence these assemblages [4]. Overall, *L. pyrotechnica* exhibits a two-tiered strategy: a diverse, environmentally responsive rhizosphere and a selective, potentially symbiotic endosphere, with Cyanobacteriota and Alphaproteobacteria as the promising bioinoculant candidates for drought resilience.

Genus-level analysis showed rhizosphere dominance by Bacillus (87–96%), with minor contributors (Noviherbaspirillum, Massilia, Cupriavidus, Kocuria, and Microvirga), matching desert soil patterns where Bacillus—notably *B. licheniformis*—enhances stress tolerance and nutrient uptake [20,21]. In contrast, stems were dominated by unclassified Cyanobacteria (~78%) and Pseudomonadota (~20%), with *Paracholeplasma* detected only in S6 (~2.5%). Cyanobacteria, known for nitrogen fixation, photoprotection, and desiccation resistance, are frequent endophytes in arid plants [22], while *Paracholeplasma* suggests novel host–microbe associations. This clear partitioning—Bacillus-rich rhizosphere and Cyanobacteria-rich stem endosphere—mirrors patterns in other desert-adapted plants, reflecting niche-specific microbial roles essential for survival in extreme environments.

Heatmap analysis revealed that clear phylum-level niche differentiation in *L. pyrotechnica*. Bacillota were highly abundant in rhizosphere samples (S1 and S4), consistent with their role in desert soils, while Cyanobacteriota dominated stem endosphere samples (S5, S6, and S7). Pseudomonadota occurred moderately across all samples, and Mycoplasmatota showed a unique increase in S6, suggesting localized or host-driven recruitment. These patterns align with desert microbiome studies, where Firmicutes (Bacillota) dominate rhizospheres due to spore-forming and stress tolerance, whereas Cyanobacteria thrive in internal tissues, likely contributing to nitrogen fixation and photoprotection [7,17].

The genus-level heatmap findings further support this niche stratification: *Bacillus* species were predominant in rhizosphere samples, aligning with their documented capacity to aid nutrient cycling and promote stress tolerance in arid environments [16], while unclassified Cyanophyceae and Alphaproteobacteria genera dominated endosphere compartments, reinforcing their probable roles in metabolic symbiosis and host adaptation under desert stress [23]. The detection of *Paracholeplasma* in S6 adds to evidence of niche-specific Mollicutes associations in plants.

Overall, the analysis confirmed a strong phylum-level shift between rhizosphere (Bacillota > 90%, moderate Pseudomonadota, Actinomycetota) and endosphere (Cyanobacteriota ~78%, Pseudomonadota ~20%, Mycoplasmatota ~2.5%) communities. Minor phyla (e.g., Bacteroidota and Gemmatimonadota) appeared only in rhizospheres, while archaea were absent. Similar compartmentalization has been reported in *Alhagi sparsifolia*, *Tamarix ramosissima*, and *Calligonum caput-medusae* [13]. In *Lantana camara* rhizospheres and similar desert species, Shannon values frequently exceed five, with diversity tapering within endospheric compartments [24]. Moreover, community clustering (via PCA) distinctly separates high-diversity rhizosphere (S1/S4) from low-diversity endosphere clusters (S5–S7), replicating patterns seen across desert ecosystems. These findings support a conserved desert plant microbiome structure: a taxonomically rich, versatile rhizosphere compared to a specialized, low-diversity endosphere enriched in phototrophic and protective taxa, shaped by soil heterogeneity and host-driven filtering.

Rarefaction analysis using ASVs, Faith’s PD, Shannon, and Gini–Simpson indices revealed a clear richness gradient in *L. pyrotechnica*. Rhizospheric samples (S1, S4) showed high richness (~530 and ~190 ASVs) and broad phylogenetic diversity (PD ≈ 45, ≈29), while endosphere samples (S5–S7) had markedly lower richness (≤33 ASVs) and PD (≈3–9). These trends mirror arid plant microbiomes, where rhizospheres host complex communities and endospheres are more selective [13,25]. Plateauing curves confirmed sufficient sequencing depth, indicating robust diversity estimates.

Beta-diversity metrics showed strong compartmentalization. Rhizospheres (S1, S4) were moderately dissimilar (Bray–Curtis dissimilarity of ~0.455) but nearly completely distinct from endosphere samples (≥0.9996). Endospheres (S5–S7) were clustered tightly (0.103–0.197 dissimilarity). The dendrogram confirmed two main clusters—rhizosphere and endosphere—with S6 and S7 being especially congruent, followed closely by S5. These findings align with patterns observed in desert plant microbiome studies. For instance, rhizosphere and root endosphere communities of *Alhagi sparsifolia* formed distinct clusters with high pairwise dissimilarity, whereas endosphere samples were more similar to each other than to rhizosphere samples [13]. Similarly, research on desert leguminous shrubs demonstrated significant niche filtration, with distinct clustering of rhizosphere and root microbiomes in both PCoA and hierarchical analyses [25]. In speargrass and halophyte studies from the Namib Desert, strong microbial compartmentalization was also reported; rhizosheath–root systems were clearly separated from bulk soil communities, with host-associated samples forming cohesive clusters [26].

PCoA confirmed this separation: rhizospheres (S1, S4) were fully segregated from endospheres (S5–S7) along PC1, explaining 90.04% of the variance. Notably, similar patterns have been reported for *Alhagi sparsifolia*, where root and rhizosphere communities exhibited strong beta-diversity differentiation in PCoA, with PC1 explaining over 70% of the variance and a tight clustering of endosphere samples separate from rhizosphere samples [13]. Beta-diversity metrics across multiple desert plants (e.g., *Alhagi sparsifolia*, *Tamarix ramosissima*, *Calligonum caput-medusae*) also showed that compartment-driven structuring of the root endosphere, rhizosphere, and bulk soil communities differed significantly in PCoA analyses [16]. Even in halophyte fungal communities, PCoA of the root endosphere distinctly separated fungal communities from those in rhizosphere or non-rhizosphere soil, underscoring the rhizocompartment as a primary structuring force [27]. Furthermore, studies on date palms in oasis ecosystems found that compartment structuring explained up to ~50% of Bray–Curtis dissimilarity in PCoA, with distinct ordination patterns identified from the root to the rhizosphere to bulk soil [28].

Taken together, these results suggest that the divergent community structures and biodiversity patterns observed between the rhizosphere and endosphere compartments of *L. pyrotechnica* are the outcome of multi-layered selection pressures. Environmental heterogeneity, nutrient and moisture gradients, and soil physical properties shape the rhizosphere as a taxonomically rich and functionally flexible community. In contrast, internal plant compartments act as highly selective microhabitats that support low-diversity, functionally specialized assemblages adapted to host-driven conditions. In future applications, identifying key microbial functional traits, such as metabolite production, enzyme activities, and stress mitigation pathways, could open avenues for developing targeted microbial inoculants or bioactive compounds with potential uses in agriculture and even regenerative medicine.

## 5. Conclusions

This study revealed that clear niche-specific compartmentalization of microbial communities in *L. pyrotechnica* from an arid ecosystem, with the rhizosphere (S1, S4) supporting more diverse, phylogenetically rich assemblages dominated by Bacillota, Actinomycetota, and Pseudomonadota, and stem tissues (S5, S6, and S7) and harboring less diverse communities enriched in Cyanobacteriota, Pseudomonadota, and Mycoplasmatota. These patterns likely reflect environmental filtering and host-driven selection, with the rhizosphere serving as a reservoir for nutrient-cycling and stress-tolerant microbes, and the endosphere favoring taxa with potential roles in nitrogen fixation, photoprotection, or osmoregulation. While functional roles are inferred from taxonomy, future metagenomic and culture-based studies are needed to confirm the capacities of these communities and assess their applications in agriculture, ecosystem restoration, and biotechnology.

## Figures and Tables

**Table 1:** Sequence quality control for 16S rRNA amplicon data across samples.

Sample Name	Raw Data	Adapter & Primer Trimming	Preprocessing Length Trimming	Quality Filter	QC Remain	Denoised Forward	Denoised Reverse	Merged Pairs	Non-Chimeric	ASV Length Filter	ASV Remain
**S1**	168,433	166,565	166,565	143,423	85.15%	141,083	142,049	133,917	113,999	113,999	67.68%
**S4**	162,585	160,837	160,837	136,755	84.11%	136,107	136,349	134,765	117,988	117,983	72.57%
**S5**	120,770	119,255	119,255	94,873	78.56%	94,743	94,794	94,689	89,304	89,304	73.09%
**S6**	142,409	140,769	140,769	120,196	84.40%	120,016	120,094	119,935	113,318	113,318	79.57%
**S7**	172,119	170,168	170,168	139,492	81.04%	139,330	139,399	139,219	133,133	133,133	77.35%

**Table 2:** Pairwise distance matrix showing dissimilarities in the microbial community of samples based on beta diversity analysis.

	S1	S4	S5	S6	S7
S1	0	0.455277	0.999764	0.999877	0.999644
S4	0.455277	0	0.999855	0.999879	0.999801
S5	0.999764	0.999855	0	0.118694	0.197184
S6	0.999877	0.999879	0.118694	0	0.103522
S7	0.999644	0.999801	0.197184	0.103522	0

Values close to 0 indicate greater similarity, while values close to 1 indicate greater dissimilarity. Color shading indicates the degree of similarity: lighter cells represent greater similarity (values closer to 0), while darker cells represent greater dissimilarity (values closer to 1).

## Data Availability

The raw data supporting the conclusions of this article will be made available by the author on request.

## Abbreviations

The following abbreviations are used in this manuscript:

ASVs	Amplicon sequence variants
PCoA	Principal coordinates analysis
UPGMA	Hierarchical clustering
QC	Quality control
